# Boron Tolerance in *Aspergillus nidulans* Is Sustained by the SltA Pathway Through the SLC-Family Transporters SbtA and SbtB

**DOI:** 10.3390/genes8070188

**Published:** 2017-07-21

**Authors:** María Villarino, Oier Etxebeste, Gorka Mendizabal, Aitor Garzia, Unai Ugalde, Eduardo A. Espeso

**Affiliations:** 1 Department of Cellular and Molecular Biology, Centro de Investigaciones Biológicas (CSIC), Ramiro de Maeztu 9, 28040 Madrid, Spain; villarino.maria@inia.es; 2 Department of Plan Protection, INIA, Carretera de la Coruña km. 7, 28040 Madrid, Spain; 3 Biochemistry II laboratory, Department of Applied Chemistry, Faculty of Chemistry, University of the Basque Country, 20018 San Sebastian, Spain; oier.echeveste@ehu.eus (O.E.); gomendizabal@hotmail.com (G.M.); agarcia@mail.rockefeller.edu (A.G.); unaiona.ugalde@ehu.eus (U.U.); 4 Laboratory of RNA Molecular Biology, Howard Hughes Medical Institute, The Rockefeller University, New York, NY 10065, USA

**Keywords:** filamentous fungi, stress-response, boron tolerance, morphogenesis, transcriptional regulation, SLC-family transporters

## Abstract

Microbial cells interact with the environment by adapting to external changes. Signal transduction pathways participate in both sensing and responding in the form of modification of gene expression patterns, enabling cell survival. The filamentous fungal-specific SltA pathway regulates tolerance to alkalinity, elevated cation concentrations and, as shown in this work, also stress conditions induced by borates. Growth of *sltA*^−^ mutants is inhibited by increasing millimolar concentrations of boric acid or borax (sodium tetraborate). In an attempt to identify genes required for boron-stress response, we determined the boric acid or borax-dependent expression of *sbtA* and *sbtB*, orthologs of *Saccharomyces cerevisiae bor*1, and a reduction in their transcript levels in a Δ*sltA* mutant. Deletion of *sbtA*, but mainly that of *sbtB*, decreased the tolerance to boric acid or borax. In contrast, null mutants of genes coding for additional transporters of the Solute Carrier (SLC) family, *sB*, *sbtD* or *sbtE*, showed an unaltered growth pattern under the same stress conditions. Taken together, our results suggest that the SltA pathway induces, through SbtA and SbtB, the export of toxic concentrations of borates, which have largely recognized antimicrobial properties.

## 1. Introduction

The interaction of organisms with the environment involves sensing and responding mechanisms. Filamentous fungi have developed multiple signal–transduction pathways. Some of them induce developmental responses under suboptimal growth conditions or in the presence of abiotic signals such as those involving air or light [1,2,3]. Others, such as those controlled by transcription factors (TFs) AP-1, PacC/Rim101, Crz1 or SltA/Ace1, act as principal regulatory mechanisms to minimize the effect of oxygen or nitrogen species (AP-1), control pH homeostasis (PacC/Rim101), and avoid a toxic intracellular accumulation of numerous mono and divalent cations (Crz1 and SltA/Ace1) [4,5,6,7].

**Figure 1:**
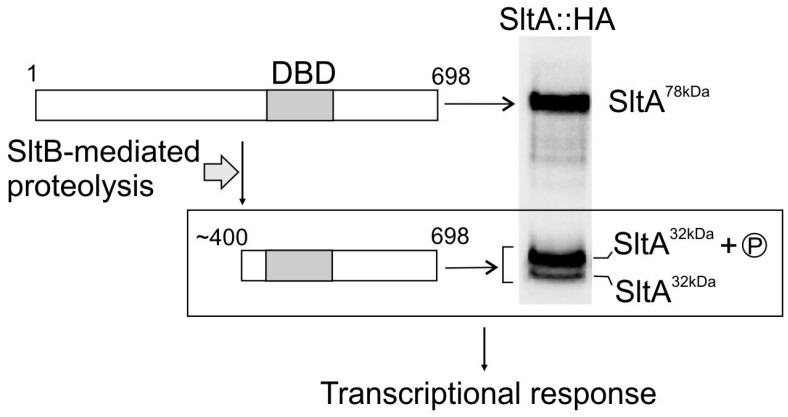
Proteolytic processing of SltA in *Aspergillus nidulans*. The full-length SltA78kDa form is proteolytically cleaved by SltB to render a truncated, active form of 32 kDa (SltA32kDa). This form comprises the DNA-binding domain (DBD) plus the poorly conserved C-terminal region, and is phosphorylated (low mobility SltA32kDa band). Shown is an immunodetection of SltA forms tagged at the C-terminus with three copies of the hemagglutinin (HA) tag, SltA::HA for brevity. Modified from [6].

SltA is a filamentous fungal-specific C_2_H_2_-type zinc finger transcription factor mediating tolerance of the model ascomycete *Aspergillus nidulans* to alkalinity and high extracellular concentrations of cations such as sodium, potassium, lithium, cesium or magnesium [8,9,10]. Different forms of SltA are found in the cell. The full-length SltA version of 78 kDa (SltA78kDa) is proteolytically processed to render a truncated, active form of 32 kDa (SltA32kDa), which starts near amino acid 400, comprises the DNA-binding domain plus a C-terminal region that is poorly conserved among homologues and is subjected to a phosphorylation step (Figure 1) [6]. Proteolysis of SltA is performed by SltB through the activity of its chymotrypsin-like-protease domain. Expression of *sltB* is positively regulated by SltA and SltB auto-proteolyzes before proteolyzing SltA [6].

Despite recent knowledge of the signaling mechanism of SltA, little is known about its transcriptional role. SltA recognizes a 5′-AGGCA sequence through its highly conserved DNA-binding domain, composed of three zinc fingers. Among genes positively regulated by SltA are *enaA*, encoding a sodium pump ATPase, as well as *pmaA* and *pmaB*, encoding vacuolar calcium ATPases [9,11]. In contrast, the vacuolar calcium exchanger-coding gene *vcxA* is negatively regulated [9]. The hypervacuolization phenotype of *sltA*^−^ mutations and their ability to suppress the strong morphological defect caused by mutations in some of the vacuolar sorting protein genes (*vps*), indicate that SltA is involved in the organization of intracellular transport and endomembranes [8,11]. SltA also participates in the transcriptional regulation of *nsdD*, *steA* and *brlA* expression, which code for key TFs of *A. nidulans* asexual and sexual development, and also in sterigmatocystin production through positive regulation of *aflR* and *stcU* genes [12]. SltA homologues have been characterized in *Hypocrea jecorina* (Ace1) and *Colletotrichum gloeosporioides* (CgSltA). Ace1 is a negative regulator of cellulase and xylanase genes [13], while CgSltA is a positive regulator of *pmk*1 and *cat*1, encoding respectively a MAP kinase and a carnitine acetyl transferase required for appressorium formation [14].

In this work, we show that SltA is also required for the tolerance of *A. nidulans* to stress conditions induced by high concentrations of borates. The addition of boric acid or borax (sodium tetraborate) to the culture medium reduced radial extension of the null and specific loss-of-function mutants of *sltA*. Since various boron-containing compounds have antimicrobial properties, we tried to identify transporters involved in the detoxification of harmful concentrations of borate and characterize their relationship with SltA. Considering that a member of the solute carrier (SLC)-family protein, Bor1, mediates the response to boron-stress in *Saccharomyces cerevisiae* [15], the phenotype of strains bearing deletions in genes coding for SLC-family proteins was analyzed. Only SbtA and SbtB, the putative orthologs of Bor1p, were required for the tolerance to boron-stress, being their transcript levels positively regulated by borate and SltA.

## 2. Materials and Methods

### 2.1. Sequence Analyses

Sequence analysis of *sbtA/An*4904, *sbtB/An*0218, *sB/An*2730 [16], *sbtD/An*3157 and *sbtE/An*3665 was done using our RNA-sequencing (RNA-seq) results [17,18] and AspGD (http://www.aspergillusgenome.org/) or FungiDB (http://fungidb.org) databases. Mapping of RNA-seq reads at corresponding *loci* was visualized using the Integrative Genomics Viewer (IGV) software (Broad Institute, Cambridge, MA, USA). Predictions of the coding region of *sbtB* made by the AspGD and FungiDB databases overlooked the presence of a processed intron within the predicted third exon, which was confirmed by RNA-seq results (Figure S1) [18,19]. This intron corresponded to a region of 21 amino acids (253–273) of SbtB, which was not considered for BLAST analyses. These were done at the National Center for Biotechnology Information NCBI (https://blast.ncbi.nlm.nih.gov/Blast.cgi) and EMBL-EBI (http://www.ebi.ac.uk/Tools/sss/ncbiblast/) websites. The sequences of predicted homologs were aligned using ClustalW (http://www.ch.embnet.org/software/ClustalW.html) or Clustal omega (http://www.ebi.ac.uk/Tools/msa/clustalo/) and visualized with open-source Genedoc, version 2.7, windows program (Pittsburg supercomputing center, Pittsburg, PA, USA). Phylogenetic trees were built using Mega, version 4.0 [20].

### 2.2. Strains, Oligonucleotides, Culture Conditions and Protoplast Transformation

The *Aspergillus nidulans* strains and oligonucleotides used in this work are shown in Tables S1 and S2. The Oligonucleotides were combined in different Polymerase Chain Reactions (PCR) in order to amplify and subsequently fuse, through fusion-PCR, 5′- and 3′-UTR regions of *sbtA*, *sbtB*, *sB*/*sbtC*, *sbtD* or *sbtE*, to *Aspergillus fumigatus* marker genes *pyrG^Af^* or *riboB^Af^* (Figure S2) [21]. Fusion–PCR constructs were used to transform protoplasts of the wild-type strains TN02A3 or TN02A25 [22]. Selection of transformants was based on *pyrG^+^* or *riboB^+^* phenotypes, depending on the selection marker (*pyrG^Af^* or *riboB^Af^*) included in the construct used to transform protoplasts. For the generation of the double null Δ*sbtA::pyrG^Af^*; Δ*sbtB::riboB^Af^* (MAD6426) strain, protoplasts of TN02A25 were first transformed so as to delete *sbtB* (using *riboB^Af^* as the selection marker, generating the strain MAD6425). In a second step, *sbtA* was deleted by transforming protoplasts of MAD6425 and using *pyrG^Af^* as the selection marker. Protoplasts were generated and transformed following standard procedures [23,24]. DNA samples for Southern-blot experiments were isolated and manipulated as described previously [25].

Strains were cultivated at 37 °C in appropriately supplemented liquid or solid (plus 1% agar) Aspergillus minimal (AMM; pH = 6.5) or complete (ACM) media, which contained glucose (1%) and ammonia (10 mM) as carbon and nitrogen sources, respectively [26]. Stress conditions were induced by adding boric acid (2, 5, 10, 15, 25, 50 or 100 mM), borax (1, 2, 5, 10 or 15 mM), L-methionine (1 mM), sodium sulfate (50 mM), thiosulfate (50 mM), sodium bicarbonate (25 mM) or potassium bicarbonate (25 mM), respectively.

For RNA extraction, the wild-type MAD2666 or MAD4097 strains and a Δ*sltA* mutant were grown in adequately supplemented liquid AMM for 18 h at 37 °C and 250 rpm. Then, boric acid (50 mM), borax (5 mM) or sodium bicarbonate (25 mM) was added to the culture medium. Samples were collected after 10, 20, 30 and 60 min of culture under the same temperature and shaking conditions (New Brunswick^TM^ Scientific Co., Inc., Edison, NJ, USA).

### 2.3. RNA Isolation and Northern-Blot Analyses

RNA samples were extracted using TriReagent (Sigma-Aldrich Quimica, Madrid, Spain) and following the manufacturer's indications. A radioactive high prime DNA labeling kit (Sigma-Aldrich Quimica) was used for the generation of probes. The hybridization solution described by Church and Gilbert was used (1% BSA, 1 mM EDTA, 0.5 M NaPO_4_, pH = 7.2 and 7% sodium dodecyl sulfate (SDS)) [27]. Hybridizations were performed overnight at 55 °C, and the filters were washed twice with a 2% saline-sodium citrate (SSC)/0.1% SDS solution at 55 °C and then with a 0.2% SSC/0.1% SDS solution, heated at the same temperature. Detection of *sbtA*, *sbtB*, *enaA* and *pho*89*^An^*^8956^ transcription was performed by exposing the blots to a PhosphorImager screen (Molecular Dynamics, GE Healthcare Europe GmbH, Freiburg, Germany) and developing using a FLA-5100 Reader (Fujifilm Life Science, FujiFilm Europe GmbH, Barcelona, Spain).

## 3. Results

### 3.1. sltA Mutants Are Less Tolerant to Boron Stress

**Figure 2:**
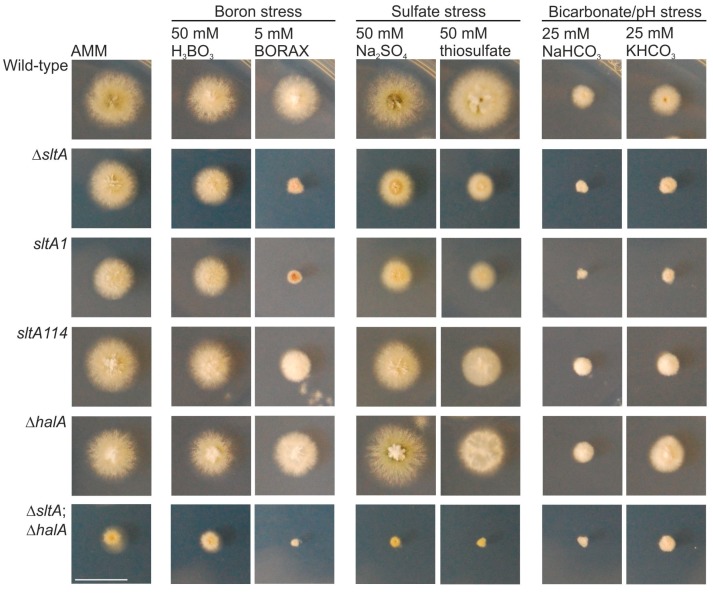
Sensitivity of loss-of-function *sltA* mutants to stress. Phenotype of wild-type, Δ*sltA*, *sltA*1, *sltA*114, Δ*halA* and Δ*sltA*; Δ*halA* strains after 48 h of cultivation at 37 °C in AMM (Aspergillus minimal medium) and AMM with 50 mM boric acid, 5 mM Borax, 50 mM sodium sulfate, 50 mM thiosulfate, or 25 mM of sodium or potassium bicarbonate. Scale bar = 2 cm.

Previous analyses showed that the SltA–SltB pathway is required for tolerance to bicarbonate and alkali metal cation stress response [8,9]. In this context, different boron-containing compounds have been described to have antimicrobial properties [28] and, thus, it would be of interest to analyze how boron homeostasis is controlled. Here a possible implication of the transcription factor SltA in response to high extracellular borate concentrations was analyzed. With that aim, the phenotypes of the null *sltA* strain and loss-of-function mutants such as *sltA*1, resulting in a truncation at amino acid 502 [10], and *sltA*114, which resulted in a Lys431Thr substitution in the first Zn finger [8], were compared to that of a wild-type strain (Figure 2).

A Δ*halA* mutant and the double-null Δ*sltA*;Δ*halA* strain were included, since previous works described the existence of a genetic interaction between *sltA* and *halA* [29]. HalA is a protein kinase required for the correct expression of vacuolar calcium permeases. Tolerance of those strains to stress conditions induced by boric acid (50 mM) or borax (5 mM) was analyzed, and the phenotypes compared to those observed under sulfate stress induced by 50 mM sodium sulfate or thiosulfate, or bicarbonate/alkaline-pH stress induced by sodium or potassium bicarbonate (25 mM). After 48 h of cultivation at 37 °C all *sltA* mutants were, as expected, more sensitive than the wild-type to the addition of bicarbonate (Figure 2). Under borate stress, these mutants, mainly null and *sltA*1 strains in medium with borax, showed a clear inhibition of radial extension, compared to the reference wild-type strain. The effect on the growth of *sltA* mutants was less severe in medium with sulfate or thiosulfate. Although borates had an almost negligible effect on the null *halA* strain, a role for this kinase in borate-, and even sulfate-, stress response cannot be ruled out due to the additive phenotype of the double null Δ*sltA*;Δ*halA* mutant.

### 3.2. SbtA and SbtB Are Required for Tolerance to Borates

**Figure 3:**
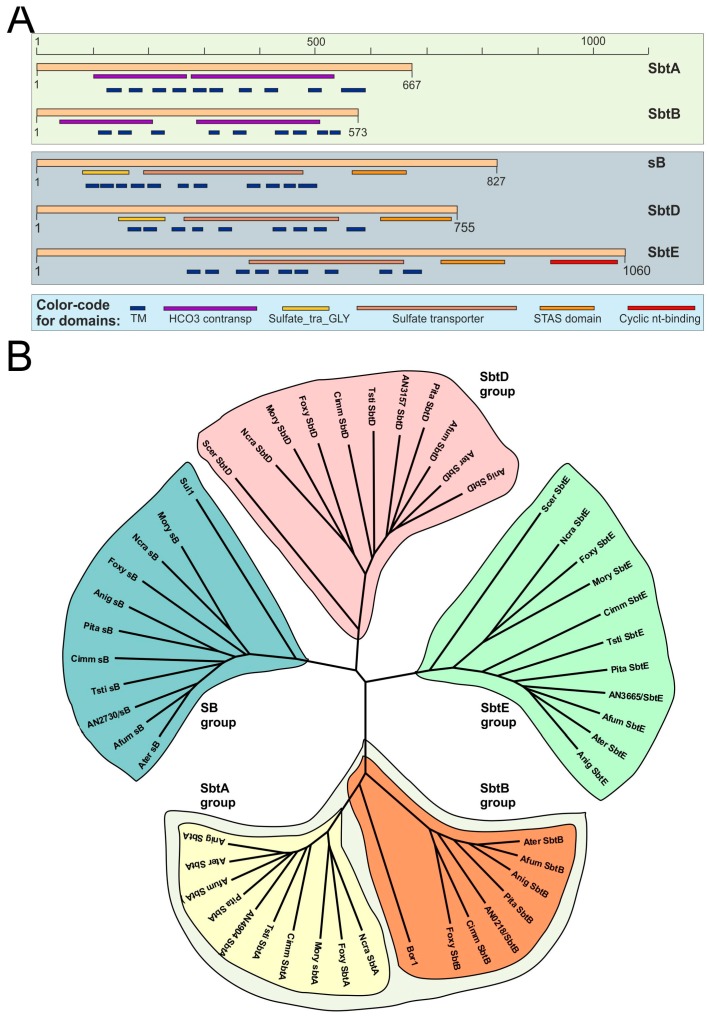
Phylogenetic analysis of *A. nidulans* Sbt proteins. (**A**) Domain organization of SbtA, SbtB, SB, SbtD and SbtE. The position and extension of predictably functional domains is indicated according to the information provided by the AspGD database. Nomenclature: Tm, transmembrane domain; HCO_3_ cotransp, bicarbonate transporter domain (IPRO11531); Sulfate_tra_GLY, sulfate transporter N-terminal domain with GLY motif (PF13792.1); Sulfate transporter, SLC26A/SulP transporter domain (IPRO11547); STAS, sulfate transporter and antisigma factor antagonist domain (IPRO02645); Cyclic nt-binding, cyclic nucleotide-binding domain (IPRO00595). This database did not predict the presence of an intron within the third exon of *sbtB*, which is spliced according to our RNA-seq results (see also Figure S1) [17,18]. This intron corresponds to a region of 21 amino acids between positions 253 and 274 of the protein sequence predicted by the AspGD. This region was not present in orthologs of SbtB and was, consequently, not considered for evolutionary analyses. (**B**) Phylogenetic tree for the ortholog groups of Sbt-s. While groups of SB, SbtD and SbtE are clearly differentiated, the conservation among SbtA and SbtB orthologs is higher (not shown) and the evolutionary distance lower. MEGA software (version 4) was used [20]. Nomenclature: An: *A. nidulans*; Afum: *Aspergillus fumigatus*; Anig: *Aspergillus niger*; Ater: *Aspergillus terreus*; Pita: *Penicillium italicum*; Cimm: *Coccidioides immitis*; Tsti: *Talaromyces stipitatus*; Ncra: *Neurospora crassa*; Foxy: *Fusarium oxysporum*; Mory: *Magnaporthe oryzae*; Scer: *Saccharomyces cerevisiae*.

Next, we tried to identify putative membrane transporters required for borate detoxification so as to subsequently analyze their relationship with SltA. Bor1p is an SLC-family protein involved in the detoxification of boron in *S. cerevisiae* [15]. We identified by BLAST searches two putative orthologs of Bor1 in *A. nidulans*, AN4904 and AN0218. Since both proteins were predicted to contain sodium bicarbonate transporter domains [30], they were named as SbtA and SbtB (Figure 3A). Three additional SLC-family proteins were identified: AN2730, which was previously characterized as SB [16], AN3157/SbtD and AN3665/SbtE. These three SLC proteins were predicted to contain sulfate transporter and STAS (sulfate transporter and antiSigma factor antagonist; [31]) domains (Figure 3A).

Coding sequences of *sbtA*, *sbtB*, *sB*, *sbtD* and *sbtE* were confirmed using our RNA-seq results [17,18], but the presence of an additional intron within the third exon of *sbtB* was observed (see Materials and Methods; see also Figure S1), making us remove 21 amino acids (253–273) from the SbtB sequence used in the BLAST and evolutionary analyses. Orthologs of all *A. nidulans* SLC-family proteins were identified in filamentous fungal classes, as for example, Eurotiomycetes and Sordariomycetes; also in Saccharomycetes such as *S. cerevisiae* and higher eukaryotes such as *Homo sapiens* or *Arabidopsis thaliana* (with the exception of SbtE, which apparently has no ortholog in *H. sapiens*) (Table S3). As occurred with Bor1p, the presence of a common ortholog of SbtA and SbtB was predicted in filamentous fungal species such as *Talaromyces stipitatus* (TSTA_032150), *Neurospora crassa* (NCU01480) or *Magnaporte oryzae* (MGG_15203) (yellow background in Table S3), supporting a common origin for these two SLC proteins. Accordingly, clades corresponding to SB, SbtD and SbtE orthologs were clearly differentiated in the phylogenetic tree shown in Figure 3B while those corresponding to SbtA and SbtB orthologs are evolutionarily closer.

**Figure 4:**
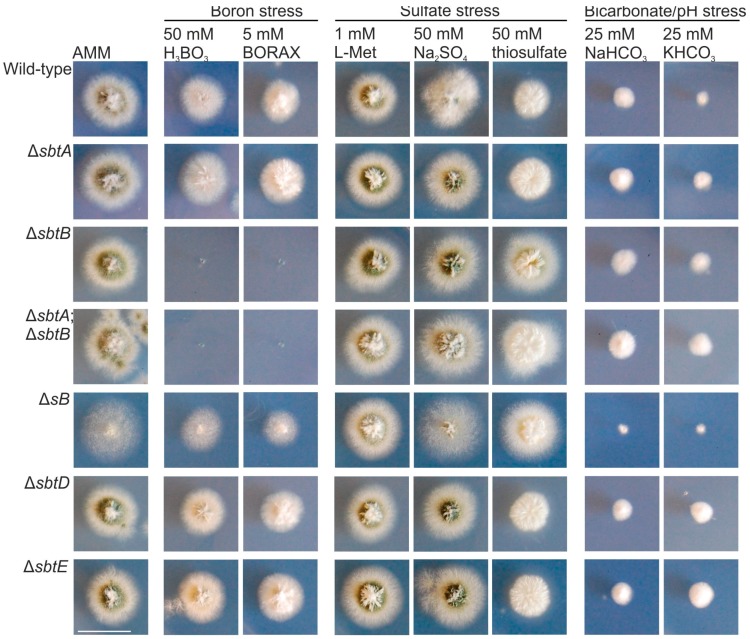
Sensitivity of *sbt* deletion mutants to stress. Phenotype of wild-type, Δ*sbtA*, Δ*sbtB*, (Δ*sbtA*;Δ*sbtB*), Δ*sB*, Δ*sbtD* and Δ*sbtE* strains after 48 h of cultivation at 37 °C in AMM and AMM with 50 mM boric acid, 5 mM Borax, 1 mM L-methionie, 50 mM sodium sulfate, 50 mM thiosulfate, or 25 mM of sodium or potassium bicarbonate. Scale bar = 2 cm.

To assess the participation of these putative five transporters in borate, sulfate, or bicarbonate/alkaline-pH tolerance, we generated their null mutants and analyzed their phenotypes under the same stress conditions shown in Figure 2 (the effect of 1 mM L-methionine was also analyzed based on the phenotypic tests done by Piłsyk and colleagues with the null *sB* strain; [16]) (Figure 4). All strains showed the same phenotype as the wild-type strain under the sulfate-stress conditions analyzed, while only Δs*B* showed a clear inhibition of growth in medium with 25 mM bicarbonate (middle and right block of images in Figure 4, respectively). The null *sB* strain showed a slight decrease in radial growth under boron stress, with an apparent lower density of hyphae (left block of images in Figure 4) [16]. Δ*sbtD* and Δ*sbtE* strains showed a wild-type phenotype under borate stress. The growth of the null *sbtB* strain (also that of the double null Δ*sbtA*;Δ*sbtB*) was completely inhibited in AMM with 50 mM boric acid or 5 mM borax, while no clear phenotypic difference was observed between the wild-type and the Δ*sbtA* mutant.

**Figure 5:**
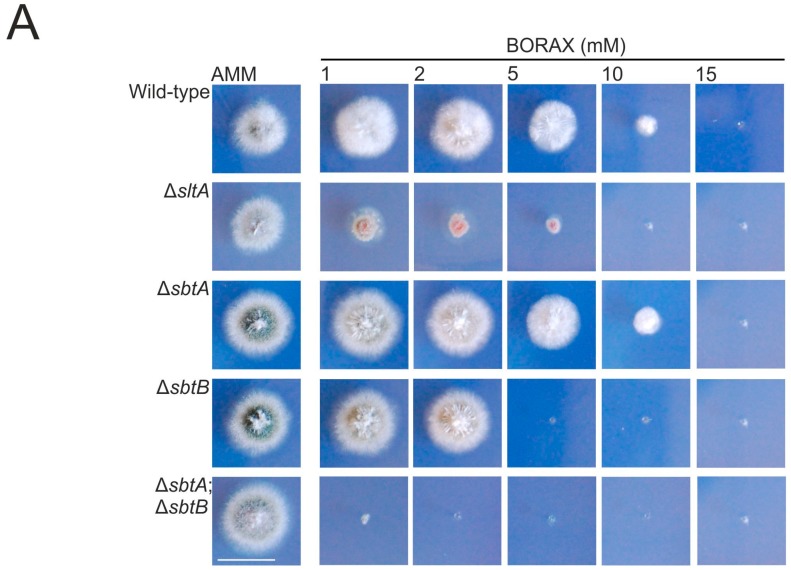

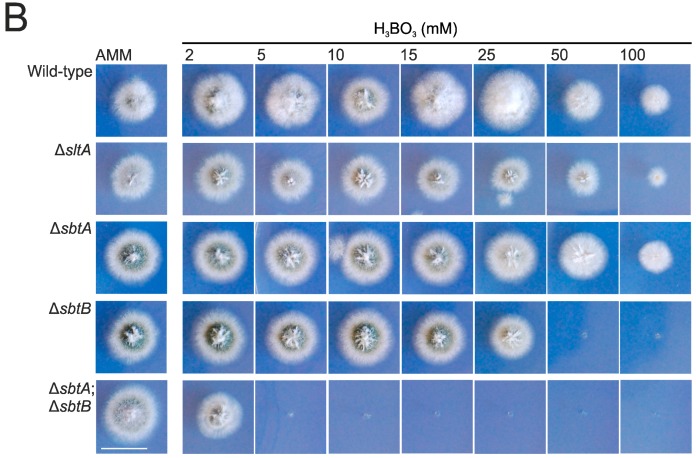
Tolerance of *sbtA* and *sbtB* deletion mutants to stress conditions induced by borates. Phenotype of wild-type, Δ*sltA*, Δ*sbtA*, Δ*sbtB* and (Δ*sbtA*;Δ*sbtB*) after 48 h of culture at 37 °C in AMM with increasing concentration of Borax (1–15 mM) (**A**) or boric acid (2–100 mM) (**B**). Scale bar in both panels = 2 cm.

Nevertheless, considering the high amino acid sequence conservation between both SLC-family proteins, we decided to proceed with a deeper characterization of the phenotypic effect that the addition of borax (1–15 mM) or boric acid (2–100 mM) to the culture medium had on these strains (Figure 5A,B, respectively). No variation in the pH value of the culture medium was observed at the end of the culture time, suggesting that the phenotypic features that will be described were not due to alkalinization. After 48 h of cultivation at 37 °C, we confirmed that the minimum boric acid or borax concentrations required for the total inhibition of the growth of the Δ*sbtB* strain lay between 26–50 mM and 3–5 mM, respectively. Again, Δ*sbtA* showed no inhibition of radial growth compared to the wild-type. However, a role for SbtA in borate-stress response can be suggested, since the double-null Δ*sbtA*;Δ*sbtB* mutant showed severe growth inhibition compared to the Δ*sbtB* strain. Growth of the double-null was completely inhibited with concentrations of boric acid or borax as low as 5 mM and 1 mM, respectively (Figure 5). Overall, it seems that the role of SbtA in tolerance to borate stress is complementary to that of SbtB. Of note, in AMM amended with borax, the Δ*sltA* strain showed a more limited radial extension than the single Δ*sbtB* strain, but not more than that of the double null Δ*sbtA*;Δ*sbtB* mutant, suggesting that additional factors may control the expression or activity of these transporters.

### 3.3. The Expression of sbtA and sbtB Is Induced Under Borate Stress and Depends on the Regulatory Activity of SltA

**Figure 6:**
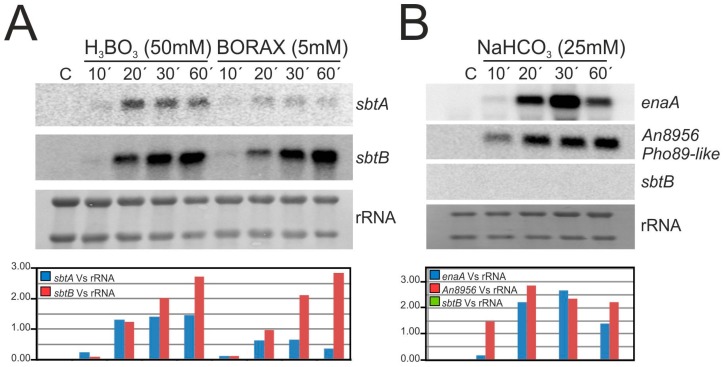

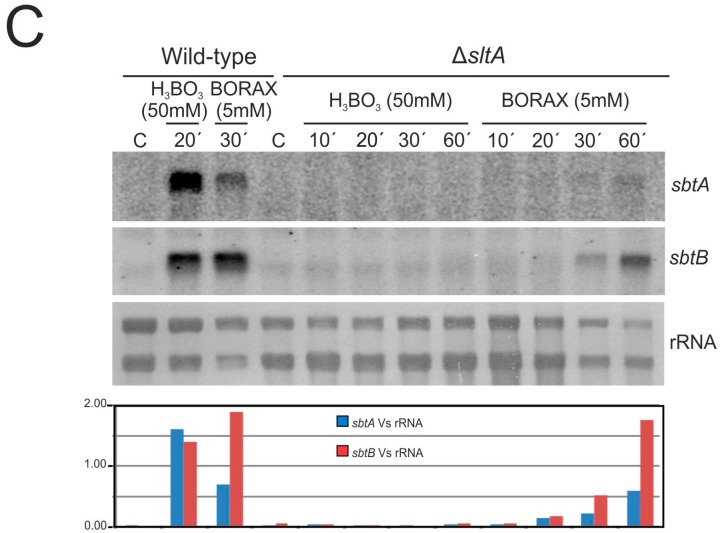
Transcriptional control of *sbtA/B* expression by SltA. Northern-blot experiments showing: (**A**) expression of *sbtA* and *sbtB* in a wild-type strain after 10, 20, 30 or 60 min of cultivation in medium with 50 mM boric acid or 5 mM Borax, (**B**) null expression of *sbtB* in a wild-type background after 10, 20, 30 or 60 min of culture in medium with 25 mM sodium bicarbonate (*enaA* and *An*8956, two of the most upregulated genes in medium with bicarbonate, were used as positive controls), and (**C**) downregulation of *sbtA* and *sbtB* expression in medium with boric acid (50 mM) or Borax (5 mM) in a Δ*sltA* background. Ribosomal RNA (rRNA) levels are shown as loading controls in all panels. The graphs below each Northern-blot show the ratios between the average pixel intensity (quantified using open-source ImageJ software (National Institutes of Health, Bethesda, MA, USA) of each Northern-blot band and the corresponding rRNA (large subunit) band.

To analyze whether the transcription of *sbtA* and *sbtB* depends on the regulatory activity of SltA and on the presence of borates in the medium, Northern-blot experiments were performed (Figure 6). First, expression of *sbtA* or *sbtB* was analyzed in AMM (control) and compared to that in AMM supplemented with 50 mM boric acid or 5 mM borax (Figure 6A). Samples were collected before and after 10, 20, 30 and 60 min of the addition of these compounds.

Results showed that the expression of both genes was detected ten minutes after the addition of boric acid or borax, and that in general (with the exception of *sbtA* in medium with borax) transcript levels increased with the time of culture under borate-stress (see quantification below the Northern-blot in Figure 6A). *sbtB* levels seemed to be higher than those of *sbtA* in almost all time-points analyzed, which correlated with the previously mentioned hypothesis that SbtB apparently has a more important role in tolerance to borates than SbtA. Transcription of *sbtB* was undetectable when mycelia were cultured in AMM supplemented with 25 mM sodium bicarbonate, while in the expression of controls *enaA* and *An*8956—the latter predictably coding for an inorganic phosphate transmembrane transporter similar to Pho89 of *S. cerevisiae*—were strongly upregulated (Figure 6B), as confirmed by RNA-seq experiments. These results support the idea that SbtB (and probably SbtA) has no role in the response to bicarbonate/pH stress.

Finally, we compared the transcript levels of *sbtA* and *sbtB* in wild-type and Δ*sltA* strains by culturing mycelia in AMM without or with 50 mM boric acid or 5 mM borax (Figure 6C). We concluded that SltA has a major role in the control of the transcription of both genes, since in the null *sltA* mutant *sbtA* and *sbtB* levels were completely inhibited in AMM with boric acid. Expression was delayed but not abolished when borax was used, suggesting that additional regulatory factor(s) might participate in controlling the expression of *sbtA* or *sbtB* under these stress conditions (see Discussion).

## 4. Discussion

Since ancient times, civilizations have used sulfates, carbonates and borates as useful chemicals for cleansing and food preservation, or even as desiccators during the mummification process [32]. Boron-derived compounds have been widely used due to both its positive and negative effects on organisms (reviewed in [33]). Boron is beneficial, sometimes essential, for plant growth and animal health, and toxicity merely depends on the levels at which it is accumulated in tissues [15].

In nature, boron is mainly found as boric acid [34]. Environmental levels and transport are two main factors of boric acid toxicity for plants and microorganisms. Compared to *A. thaliana* seeds, *S. cerevisiae* cells tolerate at least a nine times higher concentration of boric acid (10 mM versus 90 mM, respectively) ([15] and references therein). Meanwhile in *S. cerevisiae* the Bor1p transporter has been identified as a key factor in detoxifying the yeast cytoplasm of borate [35], there is little knowledge of the mechanisms of boron homeostasis in filamentous fungi. In this work we have identified two putative borate exporters. *sbtA* and *sbtB* genes encode highly similar intramembrane proteins containing domains previously defined to participate in bicarbonate transport across the plasma membrane. Similarity searches and functional studies described here support that SbtA and SbtB are both putative homologues of Bor1p of *S. cerevisiae*. Interestingly, SbtA, SbtB and Bor1p present high similarity to *Arabidopsis thaliana* BOR1 and animal bicarbonate transporters (such as NaBC1, an eletrogenic Na+-coupled borate transporter also involved in boron homeostasis) [35,36]. Bicarbonate has been found to modulate colonial growth in *A. nidulans* [37]. However, single or double null *sbtA* and *stbB* strains do not exhibit colonial growth defects that could be associated to intracellular toxic levels of bicarbonate or the accumulation of bicarbonate salts in the medium.

The genes *sbtD* and *sbtE* encode predicted transmembrane proteins related to the sulfur transporter SB, which was previously characterized in *A. nidulans* as a protein required for the uptake of diverse sources of sulfur [16]. Although SbtD, SbtE and SB proteins share a common sulphur transport (STAT) domain, null *sbtD* or *sbtE* mutants do not display the same phenotype as the Δ*sB* strain. This strain was described to show limited growth, with an apparently lower hyphal density, on AMM plates lacking an additional source of sulphur or in the presence of sulfate, but exhibited normal growth in the presence of L-methionine [16]. In contrast, null *sbtD* and *sbtE* strains grew as the wild-type in all tested conditions, including those with toxic levels of borate and bicarbonate salts. Although putative homologues of SbtD and SbtE were found in all fungal species searched, including *S. cerevisiae*, their role remains unknown.

This work is the first characterization of the mechanism that makes *A. nidulans* tolerant to high borate concentrations. A wild-type strain showed a moderate reduction in colonial growth when cultured with a 100 mM concentration of boric acid. The addition of 10 mM borax had a stronger inhibitory effect on growth, indicating that this boron compound is more toxic. Considering that *A. thaliana* is unable to germinate at 10 mM borate and that *A. nidulans* can deal with borate (boric acid) concentrations as high as 100 mM, this model fungus could be included within the group of high-boron-tolerant organisms, as occurs with *S. cerevisiae* [15]. Bor1p is responsible for boron tolerance in yeast, and as occurs with its null mutant [15,35], susceptibility of *A. nidulans* to boric acid and borax increases when the activity of its ortholog transporter, SbtB, is abrogated. Nevertheless, deletion of both *sbtA* and *sbtB* increased the sensitivity of *A. nidulans* to borate concentrations as low as 1 mM borax or 5 mM boric acid. This suggests that both putative borate exporters act coordinately in detoxifying the intracellular medium of borate excess, with SbtB having a more central role. Since homologues of SbtA and SbtB transporters can be found in most (but not all) of the fungal proteomes analyzed in this work, and both are orthologs of Bor1p from *S. cerevisiae*, it can be hypothesized that: (1) *sbtA* and *sbtB* arose by duplication, and (2) this dual exporter system may be a general mechanism of borate detoxification in multiple filamentous fungal species.

OsPIP2;4 and OsPIP2;7 are rice genes encoding boron transporters [38]. Both are upregulated in soil or media with an excess of boron. Similarly, our transcriptional studies show that *sbtA* and *sbtB* are upregulated when either boric acid or borax are added to the culture medium. *sbtA* and *sbtB* transcripts were not detected in the standard Cove AMM, which contains 40 mg/L (0.2 mM) of sodium tetraborate [26]. Addition of 50 mM boric acid or 5 mM borax allowed the detection of *sbtA* and *sbtB* transcripts as soon as 10 min after stress induction, indicating that transcriptional regulation is a major element for borate tolerance in this fungus. These results contrast with the absence of boron-dependent induction of *BOR*1 transcription in *S. cerevisae* [39]. Consequently, posttranslational modifications were proposed as the main mechanism for the control of Bor1p activity upon boron insult at the plasma membrane [35].

This work shows that SltA plays a key role in the transcriptional control of borate tolerance. The sensitivity of loss-of-function *sltA* mutant strains to boric acid and borax is probably due to the inability to induce the expression of *sbtA* and *sbtB*, as shown by Northern-blot assays. The phenotype of *sltA* mutants has been repeatedly analyzed in several genetic backgrounds and has always been consistent (see our previous references on SltA). The same occurs with the deletion of *sbtB* (it has been carried out in this work at least in two genetic backgrounds, TN02A3 and TN02A25) and the described sensitivity to boric acid and borax. Overexpression of *sltA* may result in an upregulation of *sbtA* and *sbtB* expression but we have always failed when trying to increase *sltA* levels using either constitutive or inducible promoters (not shown). Thus, we concluded that *sltA* overexpression is lethal, hampering the analysis of its effects on the levels of the two borate transporters. Rather than generating *sbtA* or *sbtB* overexpression strains by genetic recombination, which would probably have a low impact on the understanding of their physiological roles and their general regulatory mechanisms, mutants showing a constitutive or conditional (i.e., thermo-sensitive) increase in *sbtA* and *sbtB* transcript levels should be isolated. This could further the identification of a borate-specific activator and/or gain-of-function mutations in SltA which have not been isolated to date. Our previous works have shown that SltA controls the expression of target genes by binding 5′-AGGCA-3′ sequences within their promoters [9,11,12]. Promoters of *sbtA* and *sbtB* contain four SltA-target sequences each (not shown); thus, this transcriptional regulator could exert a direct effect on the expression of both genes. However, *sbtA* and *sbtB* expression is delayed but not completely inhibited in AMM with borax, suggesting that there are additional factors controlling their expression after an initial SltA-mediated fast response. For example, boron stress activates the expression and increases protein levels of the transcriptional regulator of amino acid synthesis Gcn4 (CpcA in *A. nidulans*), and the null gcn4 strain showed increased sensitivity to boric acid [40]. Similarly, the requirement of additional yeast membrane transporters for the response to boron stress have been described [15,41]. Future studies will also focus on the identification of additional regulators and transporters involved in *A. nidulans* tolerance to borates and the analysis of their functional or transcriptional relationship with SltA.

## 5. Conclusions

Our work identifies members of the SLC-family of transporters in *A. nidulans*, the Sbt transporters. Among them we focused in SbtA and SbtA which we characterize as homologs to *S. cerevisiae* Bor1p and exporters of borate. SbtA and SbtB participate in the tolerance of *A. nidulans* to elevated concentrations of borate compounds. Presence of boric acid or borax in the medium induces the expression of *sbtA* and *sbtB*. Importantly, we identified the transcriptional factor SltA, which mediates tolerance to alkaline pH and cations, as an important regulatory element required for a proper expression pattern of *sbtA* and *sbtB* genes.

## Supplementary Materials

The following are available online at www.mdpi.com/2073-4425/8/7/188/s1. Figure S1: RNAseq results for *sbtB/AN*0218. Figure S2: Procedure followed for the generation of null mutants of *Aspergillus nidulans*. Table S1: Strains used in this work. Table S2: Oligonucleotides used in this work. Table S3: Orthologs of SbtA, SbtB, SB, SbtD and SbtE in filamentous fungal species, yeast and higher eukaryotes.
