# Supplementary material for: Boron Tolerance in Aspergillus nidulans Is Sustained by the SltA Pathway Through the SLC-Family Transporters SbtA and SbtB
Source: Genes (Basel). 2017 Jul 21;8(7):188. doi: 10.3390/genes8070188 (PMC5541321; doi:10.3390/genes8070188)
Supplement: Supplementary file 1 [file genes-08-00188-s001.pdf]

**Supplementary Material:**

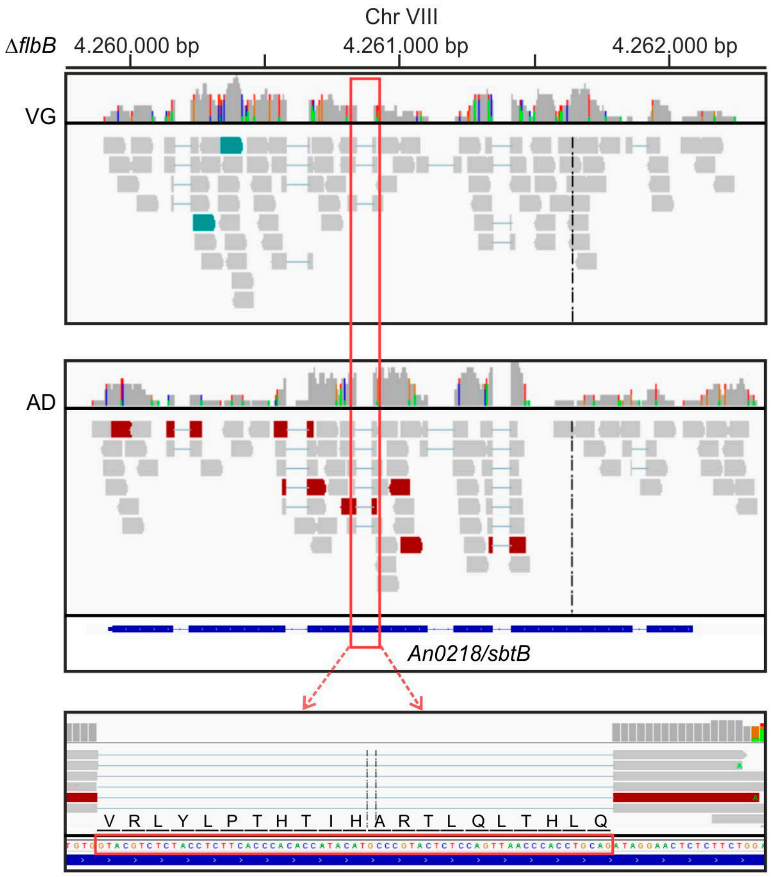


**Figure S1.** RNAseq results for *sbtB/AN0218*. Upper and lower images show mapping of reads corresponding to a Δ*flbB* strain during vegetative growth (VG) or five hours after the induction of asexual development (AD) [1]. IGV (Integrative genomics viewer) software [2] was used. The lower image corresponds to an amplification of the intronic region within exon 3 not predicted by the AspGD database. The sequence of the 21 amino acid-long region that would derive from this intron is also shown.

A

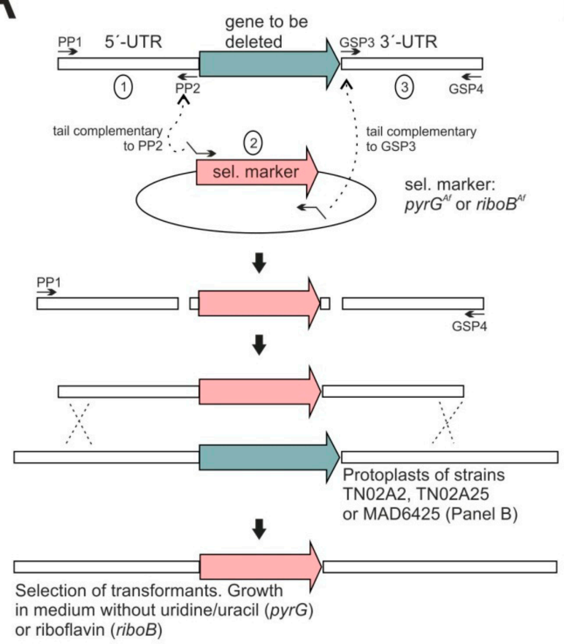


B

| Strain generated | Cassette | Recipient strain |
|---|---|---|
| BD533 | Δ*sbtA::pyrG*$^{Af}$ | TN02A3 |
| BD535 | Δ*sbtD::pyrG*$^{Af}$ | TN02A3 |
| BD536 | Δ*sbtE::pyrG*$^{Af}$ | TN02A3 |
| BD537 | Δ*sbtB::pyrG*$^{Af}$ | TN02A3 |
| MAD6424 | Δ*sB::pyrG*$^{Af}$ | TN02A25 |
| MAD6425 | Δ*sbtB::riboB*$^{Af}$ | TN02A25 |
| MAD6426 | Δ*sbtA::pyrG*$^{Af}$ | MAD6425 |

**Figure S2.** Procedure followed for the generation of null mutants of *Aspergillus nidulans*. A) The 5´- and 3´-UTR regions of the targeted gene plus the selection marker were amplified in three independent PCR reactions. In the second PCR reaction, the selection marker (*pyrG*$^{Af}$ or *riboB*$^{Af}$, *pyrG* or *riboB* genes from *A. fumigatus*) incorporated tails complementary, respectively, to the end and beginning of 5´- and 3´-UTR regions. The three fragments were fused in a fourth PCR reaction (fusion-PCR; [3]). The construct was used to transform protoplasts of recipient strains TN02A3, TN02A25 [4] or MAD6425. Selection of transformants was done based on their ability to grow on medium without uridine and uracil (*pyrG*$^{Af}$ as selection marker) or without riboflavin (*riboB*$^{Af}$ as selection marker). B) Table showing the strains generated in this work, the fusion-PCR constructs used to generate them and the corresponding recipient strains. See also Table S1.

**Table S1.** Strains used in this work.

| Strain | Genotype | Reference |
|---|---|---|
| BD533 | *pyrG89; argB2, ΔsbtA::pyrG*$^{Af}$*; pyroA4, ΔnkuA:: argB* | This work |
| BD535 | *pyrG89; argB2; pyroA4, ΔnkuA::argB; ΔsbtD::pyrG*$^{Af}$ | This work |
| BD536 | *pyrG89; ΔsbtE::pyrG*$^{Af}$*; argB2; pyroA4, ΔnkuA::argB* | This work |
| BD537 | *pyrG89; argB2; pyroA4, ΔnkuA:: argB; ΔsbtB::pyrG*$^{Af}$ | This work |
| HHF27a | Prototrophic wild type | [5] |
| HHF27b | *ΔsltA::riboB*$^{Afum}$ | [5] |
| MAD1685 | *inoB2; ΔsltA::riboB*$^{Af}$ | [6] |
| MAD1686 | *ΔhalA::pyr4*$^{Nc}$*; inoB2* | [6] |
| MAD2666 | argB2; *pyroA4,* **Δ***nkuA::argB* | [7] |
| MAD2757 | *wA3; ΔhalA::pyr4*$^{Nc}$*; inoB2; ΔsltA::riboB*$^{Af}$ | [6] |
| MAD4048 | *pyrG89, pabaA1; sltA1* | [8] |
| MAD5423 | *inoB2; ΔnkuA::BAR?; sltA114; riboB2* | [8] |
| MAD6424 | *pyrG89; argB2; ΔnkuA::argB, pabaB22; ΔsB::pyrG*$^{Af}$*; riboB2* | This work |
| MAD6425 | *pyrG89; argB2; ΔnkuA::argB, pabaB22; ΔsbtB::riboB*$^{Af}$*, riboB2* | This work |
| MAD6426 | *pyrG89; argB2, ΔsbtA::pyrG*$^{Af}$*; ΔnkuA::argB, pabaB22; ΔsbtB::riboB*$^{Af}$*, riboB2* | This work |
| TN02A3 | *pyrG89; argB2; pyroA4, ΔnkuA::argB* | [4] |
| TN02A25 | *pyrG89; argB2; ΔnkuA::argB, pabaB22; riboB2* | [4] |

All strains carry *veA1* mutation. *BAR*, herbicide bialaphos resistance gene from *Streptomyces hygroscopicus*

**Table S2.** Oligonucleotides used in this work.

| Name | Sequence (5′-3′) | Objective |
|---|---|---|
| sbtA-PP1 | GGC CAA CCA GTC CGT CG TAT CG | *sbtA* promoter |
| sbtA-PP2 | CAT GTT GAC GCT GCG GTA GTT CGA G | *sbtA* promoter |
| sbtA-GSP3 | TAA TCG ACA CGT AGA CGA TGC ACA TCC C | *sbtA* terminator |
| sbtA-GSP4 | GGC AAC CCT AGC TTC GAG ATT GAC C | *sbtA* terminator |
| sbtA-SMP1 | CTC GAA CTA CCG CAG CGT CAA CAT GAC CGG TCG CCT CAA ACA ATG CTC T | $pyrG^{Af}$ SM for *sbtA* null |
| sbtA-GFP2 | GGG ATG TGC ATC GTC TAC GTG TCG ATT AGT CTG AGA GGA GGC ACT GAT GCG | $pyrG^{Af}$ SM for *sbtA* null |
| sbtB-PP1_MVP | TGG GTG TGA TAC TCT ATT TGC G | *sbtB* promoter |
| sbtB-PP2 | CAT TGG GGA AGA ATC GAA CAT GGT C | *sbtB* promoter |
| sbtB-GSP3 | TGA AAG GAG CAG ACC CTA GGC AGA GG | *sbtB* terminator |
| sbtB-GSP4_MVP | TCA CCT GCA ACC ACA GAG C | *sbtB* terminator |
| sbtB-SMP1 | ACC ATG TTC GAT TCT TCC CCA ATG ACC GGT CGC CTC AAA CAA TGC TCT | $pyrG^{Af}$ or $riboB^{Af}$ SMs for *sbtB* nulls |
| sbtB-GFP2 | CCT CTG CCT AGG GTC TGC TCC TTT CAG TCT GAG AGG AGG CAC TGA TGC G | $pyrG^{Af}$ or $riboB^{Af}$ SMs for *sbtB* nulls |
| sB-PP1 | GGG GAG GTT GAA GTC GTC GTC GTA ATT G | *sB* promoter |
| sB-PP2 | CAT GGT GCG TTG GTG TAA TCC AAA AAC TG | *sB* promoter |
| sB-GSP3 | TGA TTG TGA TCT CCC TGG ATG GAG G | *sB* terminator |
| sB-GSP4 | CAA GGC TAC GTG AAT GTC GAG TTC CGC | *sB* terminator |
| sB-SMP1 | CTG TTT TTG GAT TAC ACC AAC GCA CCA TGA CCG GTC GCC TCA AAC AAT GCT CT | $pyrG^{Af}$ SM for *sB* null |
| sB-GFP2 | CCT CCA TCC AGG GAG ATC ACA ATC AGT CTG AGA GGA GGC ACT GAT GCG | $pyrG^{Af}$ SM for *sB* null |
| sbtD-PP1 | CTA GTC AAG ACA TAG AGC GGA GGA GCA ACC | *sbtD* promoter |
| sbtD-PP2 | CAT CGT GGC GAG CGG CG | *sbtD* promoter |
| sbtD-GSP3 | TGA GGT GCG GCT TTG GTC AGT C | *sbtD* terminator |
| sbtD-GSP4 | CAA GCC CAT ACC ACC TCC TCA ACA CAT C | *sbtD* terminator |
| sbtD-SMP1 | CGC CGC TCG CCA CGA TGA CCG GTC GCC TCA AAC AAT GCT CT | $pyrG^{Af}$ SM for *sbtD* null |
| sbtD-GFP2 | GAC TGA CCA AAG CCG CAC CTC AGT CTG AGA GGA GGC ACT GAT GCG | $pyrG^{Af}$ SM for *sbtD* null |
| sbtE-PP1 | GTA CCA AAC CGA GCT TGT CCG | *sbtE* promoter |
| sbtE-PP2 | CAT AGC TGT CTA GGC ACG AAT AGA AGG GG | *sbtE* promoter |
| sbtE-GSP3 | TGA GTC CTT CCC ACC ACC ACC | *sbtE* terminator |
| sbtE-GSP4 | GCG GGC TTA AAT AGG CGT TGC TG | *sbtE* terminator |
| sbtE-SMP1 | CCC CTT CTA TTC GTG CCT AGA CAG CTA TGA CCG GTC GCC TCA AAC AAT GCT CT | $pyrG^{Af}$ SM for *sbtE* null |
| sbtE-GFP2 | GGT GGT GGT GGG AAG GAC TCA GTC TGA GAG GAG GCA CTG ATG CG | $pyrG^{Af}$ SM for *sbtE* null |
| sbtA-exon 1 | TCG TTG CAG GAA TCC TAG GC | *sbtA* probe-Northern blot |
| sbtA-exon 2 | CTC TGA TTT CCC ATG TTC TTC C | *sbtA* probe-Northern blot |
| sbtB-exon 1 | AAG CGA CGT CTG TGA CGG AGC | *sbtB* probe-Northern blot |
| sbtB-exon 2 | ACC TAG ACT CTC CAT TAC GG | *sbtB* probe-Northern blot |
| Pho89-exon1 | CAC AAC GCA GAG TAT CAT TGG | *AN8956* probe-Northern blot |
| Pho89-exon2 | TAA CGA GGA ACC ACA CTG G | *AN8956* probe-Northern blot |
| EnaA-fw 1 | TCA ACG TCG GAA CAC CTC TT | *enaA* probe-Northern blot |
| EnaA-rev 2 | TTG ACA CCG TCA CCA GTC AT | *enaA* probe-Northern blot |

SM, selectable marker.

**Table S3.** Orthologs of SbtA, SbtB, SB, SbtD and SbtE in filamentous fungal species, yeast and higher eukaryotes.

| Species | SbtA | SbtB | SB | SbtD | SbtE |
|---|---|---|---|---|---|
| *Aspergillus nidulans* | An4904<br>L: 667 | An0218<br>L: 573 | An2730<br>L: 827 | An3157<br>L: 755 | An3665<br>L: 1060 |
| *Aspergillus terreus* | ATEG_4612<br>L:645<br>Sc:988<br>E:0.0 | ATEG_05282<br>L:583<br>Sc:869<br>E:0.0 | ATEG_02570<br>L:841<br>Sc:1312<br>E:0.0 | ATEG_04052<br>L:757<br>Sc:1056<br>E:0.0 | ATEG_03129<br>L:1098<br>Sc:1473<br>E:0.0 |
| *Aspergillus fumigatus* | Y699_01463<br>L:679<br>Sc:975<br>E:0.0 | Y699_06302<br>L:585<br>Sc:853<br>E:0.0 | Y699_06792<br>L:847<br>Sc:1299<br>E:0.0 | Y699_01217<br>L:733<br>Sc:956<br>E:0.0 | Y699_04898<br>L:1070<br>Sc:1464<br>E:0.0 |
| *Aspergillus niger* | An02g06580<br>L:671<br>Sc:968<br>E:0.0 | An01g01790<br>L:543<br>Sc:861<br>E:0.0 | An15g04600<br>L:809<br>Sc:1040<br>E:0.0 | An02g09350<br>L:730<br>Sc:985<br>E:0.0 | An01g07860<br>L:1066<br>Sc:1498<br>E:0.0 |
| *Penicillium italicum italicum* | PITC_012880<br>L:630<br>Sc:955<br>E:0.0 | PITC_022700<br>L:592<br>Sc:805<br>E:0.0 | PITC_019010<br>L:835<br>Sc:986<br>E:0.0 | PITC_061370<br>L:772<br>Sc:933<br>E:0.0 | PITC_067670<br>L:1057<br>Sc:1371<br>E:0.0 |
| *Coccidioides immitis* | CIMG_13744<br>L:663<br>Sc:945<br>E:0.0 | CIMG_02924<br>L:595<br>Sc:771<br>E:0.0 | CIMG_04891<br>L:825<br>Sc:1072<br>E:0.0 | CIMG_00108<br>L:723<br>Sc:845<br>E:0.0 | CIMG_07040<br>L:1084<br>Sc:1119<br>E:0.0 |
| *Talaromyces stipitatus* | **TSTA_032150**<br>L:691<br>Sc:928<br>E:0.0 | **TSTA_032150**<br>L:691<br>Sc:492<br>E:2e$^{-165}$ | TSTA_019080<br>L:833<br>Sc:1081<br>E:0.0 | TSTA_029710<br>L:782<br>Sc:846<br>E:0.0 | TSTA_076660<br>L:1094<br>Sc:1272<br>E:0.0 |
| *Neurospora crassa* | **NCU01480**<br>L:618<br>Sc:774<br>E:0.0 | **NCU01480**<br>L:618<br>Sc:476<br>E: 4e$^{-160}$ | NCU03235<br>L:916<br>Sc:928<br>E:0.0 | NCU09642<br>L:799<br>Sc:737<br>E:0.0 | NCU02632<br>L:1118<br>Sc:900<br>E:0.0 |
| *Fusarium oxysporum* | FOYG_02892<br>L:609<br>Sc:771<br>E:0.0 | FOYG_17224<br>L:602<br>Sc:787<br>E:0.0 | FOYG_00665<br>L:787<br>Sc:920<br>E:0.0 | FOYG_12381<br>L:801<br>Sc:758<br>E:0.0 | FOYG_06967<br>L:1082<br>Sc:915<br>E:0.0 |
| *Magnaporthe oryzae* | **MGG_15203**<br>L:701<br>Sc:770<br>E:0.0 | **MGG_15203**<br>L:701<br>Sc:465<br>E:1e$^{-154}$ | MGG_04640<br>L:844<br>Sc:940<br>E:0.0 | MGG_09838<br>L:800<br>Sc:752<br>E:0.0 | MGG_04433<br>L:1103<br>Sc:923<br>E:0.0 |
| *Saccharomyces cerevisiae* | **Bor 1**<br>L:576<br>Sc:451<br>E:7e$^{-151}$ | **Bor 1**<br>L:576<br>Sc:363<br>E:5e$^{-118}$ | Sul1<br>L:859<br>Sc:526<br>E:1e$^{-172}$ | YPR003C<br>L:754<br>Sc:414<br>E:3e$^{-133}$ | YGR125W<br>L:1036<br>Sc:644<br>E:0.0 |
| *Homo sapiens* | SCL4A1<br>L:911<br>Sc:197<br>E:2e$^{-52}$ | SCL4A3-Isoform CAE3<br>L:936<br>Sc:167<br>E:8.0e$^{-43}$ | SLC26A11<br>L:606<br>Sc:248<br>E:1e$^{-70}$ | SLC26A6-Isoform 6<br>L:671<br>Sc:169.1<br>E:2e$^{-43}$ | ------------- |
| *Arabidopsis thaliana* | At4g32510<br>L:675<br>Sc:180<br>E:2e$^{-48}$ | BOR2<br>L:703<br>Sc:170<br>E:3e$^{-45}$ | SULTR3;1<br>L:703<br>Sc:213<br>E:1e$^{-58}$ | SULTR3;3<br>L:631<br>Sc:207<br>E:1e$^{-57}$ | SULTR3;5<br>L:634<br>Sc:57.0<br>E:7e$^{-08}$ |

L: Length; Sc: Score; E: E value. Yellow background indicates a common ortholog for both *sbtA* and *sbtB* in that genome.

1. Oiartzabal-Arano, E.; Garzia, A.; Gorostidi, A.; Ugalde, U.; Espeso, E. A.; Etxebeste, O. Beyond asexual development: Modifications in the gene expression profile caused by the absence of the *Aspergillus nidulans* transcription factor FlbB. *Genetics* **2015**, *199*, 1127–42.
2. Robinson, J. T.; Thorvaldsdóttir, H.; Winckler, W.; Guttman, M.; Lander, E. S.; Getz, G.; Mesirov, J. P. Integrative genomics viewer. *Nat. Biotechnol.* **2011**, *29*, 24–26.
3. Yang, L.; Ukil, L.; Osmani, A.; Nahm, F.; Davies, J.; De Souza, C. P. C.; Dou, X.; Perez-Balaguer, A.; Osmani, S. A. Rapid production of gene replacement constructs and generation of a green fluorescent protein-tagged centromeric marker in *Aspergillus nidulans*. *Eukaryot. Cell* **2004**, *3*, 1359–62.
4. Nayak, T.; Szewczyk, E.; Oakley, C. E.; Osmani, A.; Ukil, L.; Murray, S. L.; Hynes, M. J.; Osmani, S. A.; Oakley, B. R. A versatile and efficient gene-targeting system for *Aspergillus nidulans*. *Genetics* **2006**, *172*, 1557–66.
5. Findon, H.; Calcagno-Pizarelli, A.-M.; Martínez, J. L.; Spielvogel, A.; Markina-Iñarrairaegui, A.; Indrakumar, T.; Ramos, J.; Peñalva, M. A.; Espeso, E. A.; Arst, H. N. Analysis of a novel calcium auxotrophy in *Aspergillus nidulans*. *Fungal Genet. Biol.* **2010**, *47*, 647–655.
6. Mellado, L.; Calcagno-Pizarelli, A. M.; Lockington, R. A.; Cortese, M. S.; Kelly, J. M.; Arst, H. N.; Espeso, E. A. A second component of the SltA-dependent cation tolerance pathway in *Aspergillus nidulans*. *Fungal Genet. Biol.* **2015**, *82*, 116–128.
7. Garzia, A.; Etxebeste, O.; Rodríguez-Romero, J.; Fischer, R.; Espeso, E. A.; Ugalde, U. Transcriptional changes in the transition from vegetative cells to asexual development in the model fungus *Aspergillus nidulans*. *Eukaryot. Cell* **2013**, *12*, 311–321.
8. Mellado, L.; Arst, H. N.; Espeso, E. A. Proteolytic activation of both components of the cation stress-responsive Slt pathway in *Aspergillus nidulans*. *Mol. Biol. Cell* **2016**, *27*, 2598–612.
